# Unmasking Hepatitis A: A Case Study of Atypical Presentation in a Returning Traveler From Egypt

**DOI:** 10.1155/crhe/8150734

**Published:** 2025-03-06

**Authors:** Emmanuel Edwar Siddig, Claude M. Muvunyi, Ayman Ahmed

**Affiliations:** ^1^Faculty of Medical Laboratory Sciences, University of Khartoum, Khartoum, Sudan; ^2^Rwanda Biomedical Centre, Kigali, Rwanda; ^3^Institute of Endemic Diseases, University of Khartoum, Khartoum 11111, Sudan

**Keywords:** atypical lymphocytes, atypical presentation, diagnostic challenge, hepatitis A, travel medicine

## Abstract

Hepatitis, characterized by inflammation of the liver, arises from various infectious and noninfectious causes, with viral hepatitis being caused by a diverse group of viruses including hepatitis A, B, C, D, and E. Infection with the Hepatitis A virus (HAV) can result in liver inflammation and damage, primarily spread through fecal–oral contamination. Clinical symptoms often overlap with other infections, complicating diagnosis in returning travelers from endemic regions. This case study focuses on a 46-year-old Sudanese housewife who presented with symptoms of fever, chills, headache, and muscle aches, along with a high temperature of 103.5°F, following a recent visit to Egypt. The initial assessment showed hemodynamic stability and abnormal liver function tests. This raise suspicion about the potential involvement of several infections including malaria, hepatitis, arboviral diseases such as Chikungunya, Yellow, and dengue fevers. Further investigations revealed acute hepatitis A infection confirmed through positive serology. Notably, the patient displayed atypical features such as atypical lymphocytosis, splenomegaly, and mild anemia. This case emphasizes the significance of essentially considering a wide range of diseases among travelers including hepatitis A among people coming from highly endemic areas such as Egypt, even when the patient is not manifested with the typical clinical presentation of specific disease. Particular attention is needed for epidemic-prone infections like hepatitis A.

## 1. Introduction

Hepatitis or so-called inflammation of the liver can stem from various infectious and noninfectious causes (heavy alcohol consumption, toxic exposure, or immunologic) [1–4]. Viral hepatitis, caused by a diverse group of viruses including hepatitis A, B, C, D, and E, leads to liver inflammation and damage. While hepatitis A and E are typically develop acute infections, hepatitis B, C, and D can lead to both acute and chronic infections [5].

Hepatitis A virus (HAV) is mainly transmitted through the fecal–oral contamination route and occasionally via blood transfusion [6]. Unlike hepatitis B and C, there is no chronic carrier state associated with HAV. Infections with HAV are often spread epidemically rather than sporadic cases, with infected travelers playing major risk factor for the disease dynamic and re/introduction in naïve populations [1, 5, 6].

**Figure 1:**
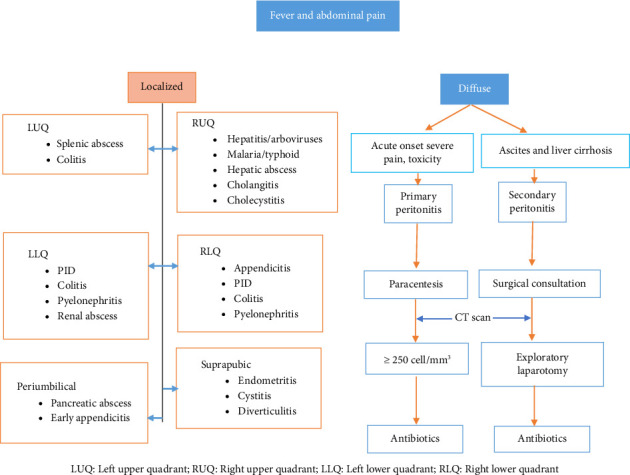
Evaluation strategy for patients presenting with fever and abdominal pain.

The clinical presentation of hepatitis A includes symptoms such as fever, malaise, nausea, vomiting, abdominal discomfort, dark urine, and jaundice [7]. For patients with a travel history presenting with fever, diagnosing hepatitis A can be challenging due to overlapping symptoms with various infections, including Yellow fever, malaria, typhoid fever, dengue fever, chikungunya, influenza, and other hepatitis viruses (Table 1) [8–10]. Therefore, to achieve an accurate final diagnosis and implement effective case management, it is crucial to conduct a comprehensive differential diagnosis supported by robust diagnostic tools. Careful consideration of current symptoms, as well as medical and travel history, can assist in distinguishing between these infections (Figure 1). Chills, for instance, are commonly associated with malaria, dengue fever, chikungunya, COVID-19, Rift valley fever, yellow fever, and influenza [11–14] (Table 1). In the case of splenomegaly, it points towards malaria and typhoid fever [13, 14]. Leukopenia is a sign often seen in dengue, chikungunya, COVID-19, or influenza infections [15–18] (Table 1). Notably, atypical lymphocytosis is characterized by unusual lymphocyte counts and is typical among malaria, dengue fever, chikungunya, and influenza infections [19, 20]; however, it is not a feature of typhoid fever. Jaundice is a common clinical manifestation for yellow fever and hepatitis [1]. Thrombocytopenia, a low platelet count, is prevalent in dengue fever but can also manifest in cases of influenza, yellow fever, and malaria [20–22]. Furthermore, observing the levels of serum transaminases in the blood can provide further insights into the potential underlying conditions. Elevated serum transaminases are particularly notable in cases of hepatitis A and E, aiding in the diagnostic process for these specific viral infections [19]. The aim of this case report is to highlight the complexities involved in diagnosing hepatitis A, particularly in patients with recent travel histories, and to emphasize the importance of thorough differential diagnosis supported by robust diagnostic tools.

## 2. Clinical Presentation

A 46-year-old Sudanese housewife was presented with fever, chills, headache, and muscle aches, accompanied by a high temperature of 103.5°F, following a 3-week visit to Egypt. Her symptoms persisted, leading to hospitalization. Upon admission, aside from myalgias and upper quadrant pain indicative of potential hepatitis, her review of systems was unremarkable. Despite being vaccinated for yellow fever, she was investigated for infection with arboviral diseases including chikungunya, yellow and dengue fevers, and malaria. She denied any history of local trauma or family members with similar conditions.

The initial clinical assessment of the patient indicated hemodynamic stability with a heart rate of 104, respiratory rate of 19, temperature of 103.5°F, blood pressure of 138/85, and oxygen saturation of 100% while breathing room air. Upon physical examination, the patient appeared well nourished and was not in acute distress. She was alert, oriented, and responding appropriately to questions, albeit with brief answers.

Her initial blood work revealed a white blood cell count of 7.400/μL, slightly low hemoglobin at 8.8 g/dL, hematocrit of 32.1%, and a normal platelet count of 390.000/μL. Liver function tests at the time of the presentation revealed a mild elevation with AST at 35 IU/L and ALT at 55 IU/L. Blood test showed 5% atypical lymphocytes with an elevated ESR of 26 mm/h. Notably, her lactate dehydrogenase (LDH) levels were significantly high at 1231 IU/L. These findings suggest several potential differential diagnoses, with viral infections being a primary consideration. Viral hepatitis, especially hepatitis A (HAV), is notable, as atypical lymphocytes can be seen in response to viral infections, and elevated LDH may indicate hepatocyte damage. Other viral infections such as hepatitis B (HBV), hepatitis C (HCV), cytomegalovirus (CMV), and Epstein–Barr virus (EBV) can also present with similar hematological changes. In addition to viral causes, severe bacterial infections or sepsis may induce a significant inflammatory response, leading to elevated ESR and LDH levels while potentially causing atypical lymphocyte presence. Tuberculosis, especially extrapulmonary cases, can affect the liver and present with elevated ESR and atypical lymphocytes. Hematological disorders, such as lymphoproliferative disorders, including non-Hodgkin lymphoma or leukemia, may cause atypical lymphocytes and elevated LDH due to increased cellular turnover.

On admission to the hospital, the presence of left upper quadrant pain prompted an abdominal CT scan, revealing mild splenomegaly. Blood samples were collected and tested for malaria and babesiosis, as well as negative blood and stool cultures for pathogenic bacterial, and her AST and ALT levels markedly increased to 478 IU/L and 521 IU/L, respectively. The patient became jaundiced and the rise of liver enzymes led to further investigation of viral hepatitis involvement. Laboratory results were negative for yellow and dengue fever, EBV, and CMV; however, it confirmed acute hepatitis A infection through markedly elevated anti-HAV IgM levels.

The patient underwent a second consultation that recommended monitoring the liver functions. On the patient's 10th day in the hospital, a follow-up hepatic function lab panel showed improvement in the patient's liver enzymes and overall liver function. Subsequently, the patient was discharged upon receiving medical clearance from the consultants, with a plan for strict clinic-based follow-up. This included thorough counseling on risk factor modification, ensuring resolution of jaundice and monitoring viral shedding for optimal recovery.

## 3. Discussion

In this communication, we report on a 46-year-old Sudanese housewife likely acquired acute hepatitis A infection during her recent visit to Egypt. She was presented with clinical symptoms including fever, chills, headache, muscle aches, right upper quadrant pain, and notably high temperature. Given her travel between Sudan and Egypt—countries known for their endemic rates of yellow fever and hepatitis viruses—this clinical picture is particularly relevant [23]. The initial blood work showed mild anemia, mild leukopenia, elevation in liver enzymes (AST and ALT), and high LDH levels. Laboratory investigation confirmed infection HAV.

This case presents several intriguing aspects that warrant attention. Initially, her clinical manifestations necessitate a comprehensive differential diagnosis, which includes hepatitis, malaria, typhoid fever, and arboviral infections, as well as potential complications such as abscesses, cholangitis, and cholecystitis (Figure 1). In addition, the serum aminotransferases were not substantially elevated, which is unusual for hepatitis A, where transaminases are typically elevated early in the course of infection. Furthermore, the presence of atypical lymphocytosis and splenomegaly, which are uncommon features of acute hepatitis A, added complexity to the diagnostic process. Unless a combination of infections was at play, the presentation of acute hepatitis A in this case was remarkably atypical. This underscores that hepatitis A should always be considered in the differential diagnosis of returning travelers from high-risk regions even if the aminotransferases show only minor elevations initially. Subsequently, the significant rise in serum transaminases during the hospital course, with AST reaching 1430 IU/L and ALT 1210 IU/L, prompted further investigation for hepatitis, ultimately confirming hepatitis A through positive serological testing.

This case is interesting for several reasons. First, HAV is commonly ignored during differential diagnosis of returning travelers, which increase the risk of introducing the disease epidemics in naïve population. Second, serum transaminases are usually highly elevated early, which was not the case here. Third, she had splenomegaly, which is not usually a feature of HAV. Furthermore, atypical lymphocytes are unusual in HAV [24, 25]. This highlights the potential for misdiagnosis, as HAV can mimic other infections. Such errors can lead to inappropriate management strategies, exacerbating disease prognosis, mortality, and the risk of further spread within the community.

In conclusion, this case underscores the critical need for a comprehensive differential diagnosis approach when evaluating patients with recent travel history to areas endemic with hepatitis A. It reinforces the importance of vigilant monitoring of liver function tests and the need to consider diverse infectious etiologies. From a public health perspective, the findings emphasize the necessity for targeted prevention and control strategies for hepatitis A, particularly among travelers to high-risk regions. Increased awareness and education regarding vaccination can play a pivotal role in safeguarding individuals against the virus. Moreover, public health initiatives should focus on monitoring and responding to potential outbreaks proactively, ensuring accessible vaccination programs in endemic areas, such as Egypt. By implementing these strategies, we can reduce the risk of hepatitis A transmission, protect vulnerable populations, and prevent the introduction of the disease into naive communities.

## 4. Patient Perspective

As a 46-year-old housewife from Sudan, my recent trip to Egypt took an unexpected turn when I developed a high fever, chills, headaches, and abdominal pain, leading me to seek medical attention. Initially, I feared a serious illness, especially in the context of various infectious diseases circulating during my travels. After thorough testing, I was diagnosed with hepatitis A, and I was worried about the implications for my recovery and the health of my family. Throughout this experience, the healthcare team provided exceptional support, helping me to understand the nature of my illness and the necessary steps for recovery. I learned valuable lessons about the importance of being vigilant regarding my health, particularly when traveling to regions where infectious diseases are common. This experience heightened my awareness of travel-related health risks and encouraged me to better educate myself about the vaccinations and preventive measures necessary before embarking on future trips. Postdischarge, my diagnosis significantly influenced my behaviors. I became more conscientious about personal hygiene, ensuring that I washed my hands frequently and avoided consuming uncooked foods and unfiltered water while traveling. In addition, I initiated discussions within my family and community about the importance of vaccination against hepatitis A and the need to be aware of health risks associated with travel. I now feel motivated to advocate for better health practices in my community, promoting awareness of contagious diseases and emphasizing the importance of preventive measures like vaccinations. My experience has shown me how crucial it is not only to protect my own health but also to contribute to the wellbeing of others by sharing knowledge on preventing the spread of infections like hepatitis A.

## Figures and Tables

**Table 1:** Differential diagnosis of acute febrile illness in travelers.

	Malaria	COVID-19	Typhoid	Dengue	Chikungunya	Influenza	Hepatitis A
*Symptoms*							
Fever	+++	+++	++	++	+++	+	+
Chills	+++	+++	−	±	±	+	−
Headache	+	++	+	++	++	++	−
Joint pain	−	++	−	+	+++	+	−
Muscle pain	+	++	−	+++	+	+++	−
Dry cough	±	++	±	−	−	+++	−
Malaise/fatigue	+	++	+	−	−	+++	+
Pruritus	−	+	−	−	+++	−	−
Encephalitis	±	±	±	++	+++	−	−
Hemorrhage	+	±	±	++	++	++	−
Jaundice	+	+	++	+++	+++	−	+++

*Signs*							
Fever > 102°F	+	+	+	+	+	±	−
Relative bradycardia	+	+	+	+	−	−	−
Conjunctivitis	−	+	−	±	−	±	−
Bilateral posterior cervical adenopathy	−	−	−	±	+	−	±
Truncal rash	−	+	±	+++	+++	−	−
Hepatomegaly	±	+	+	−	−	−	±
Splenomegaly	+	+	+	−	−	−	±
Arthritis	−	+	−	−	+++	−	−

*Laboratory tests*							
Leukopenia	−	+	−	+++	+	±	±
Relative lymphopenia	+	±	+	++	+++	++	−
Atypical lymphocytes	+	++	−	+	±	±	±
Thrombocytopenia	+	+++	−	+++	−	+	−
Highly elevated ESR	+++	+++	−	−	−	−	−
Mild/moderately elevated LDH	+++	+++	−	±	±	±	−
Mild/moderately elevated AST/ALT	+	+	+	+	+	+	++

*Note:* + = mild manifestation, ++ = moderate manifestation, +++ = severe manifestation, and − = no manifestation.

## Data Availability

The data that support the findings of this study are available from the corresponding author upon reasonable request.

## Nomenclature

ALT: Alanine aminotransferase
AST: Aspartate transferase
HAV: Hepatitis A virus
LDH: Lactate dehydrogenase
